# Supplementary material for: Hydroxyapatite Formation Coexists with Amyloid-like Self-Assembly of Human Amelogenin
Source: Int J Mol Sci. 2020 Apr 22;21(8):2946. doi: 10.3390/ijms21082946 (PMC7216246; doi:10.3390/ijms21082946)

Hydroxyapatite Formation Coexists with Amyloid-like Self-assembly of Human Amelogenin

Jing Zhang [1, 2, 3], Jian Wang [1] Cheng-wei Ma[1] and Jun-xia Lu [1,*]

[1] School of Life Science and Technology, ShanghaiTech University, Shanghai 201210, P. R. China;
[2] State Key Laboratory of Molecular Biology, CAS Center for Excellence in Molecular Cell Science, Shanghai Institute of Biochemistry and Cell Biology, Chinese Academy of Sciences, Shanghai 200031, P. R. China;
[3] University of Chinese Academy of Sciences, Beijing 100049, P. R. China
* Correspondence: lujx@shanghaitech.edu.cn;

Table S1 Chemical shift assignments for a β-sheet region (residues ^{14}N-^{31}P) of H175 assemblies formed at pH 4.5 and 6.0 conditions based on MAS NMR experiments.

Res Number	Res Type	N	CO	CA	CB	CG	CG1	CG2	CD	CD1	CD2	CE	CE2	NE	NE1	NE2	NZ
14	N	126.7		52.69	41.81												
15	F		174.6	56.66	41.8												
16	S	125.7	172.5	56.65	65.27												
17	Y	123.4															
18	E		173.9	55.31	29.43	33.64			180.2								
19	V	127.7	173.1	61.28	34.88	21.92											
20	L	130.6	176.2	53.27	44.81	28.47											
21	T	114.3	173	54.9	70.34	18.1											
22	P	137.6	174.4	62.6	32.17	27.56			48.42								
23	L	130.9	176.3	55.68	45.03	29.5				26.65	24.2						
24	K	130.7	172.8	53.63	32.66	25.1			29.23			42.31					34.92
25	W	130.1	174.5	52.95	32.65	111.1				121.9	128.6		138		127.3		
26	Y	127.4		55.71	41.68												
27	Q	120.1	174	54.8	35.78	35.83			179.1							113.6	
28	S	119.2	173.7	55.42	63.35												
29	I	128.6	174.2	59.76	40.97		27.4	17.31		13.04							
30	R			56.04	30.33	27.19			42.52					95.33			
31	P			61.96	30.78	27.3			50.04								

Table S2 Chemical shift assignments for additional rigid sites and flexible sites of H175 assemblies formed at pH 6.0. The assignment on the rigid sites is from CP-based MAS SSNMR experiments (top section of the table) while the assignment on the flexible sites is based on INEPT-TOBSY experiment (bottom half of the table). Secondary chemical shifts were also calculated. The random coil chemical shifts values used in the calculation were taken from biological magnetic resonance data bank (www.bmrb.wisc.edu/ref_info/).

Residue	Atom Type	Secondary chemical shifts (ΔCα)	Experimental chemical shifts	Random coil chemical shifts	Atom Type	Secondary chemical shifts (ΔCβ)	Experimental chemical shifts	Random coil chemical shifts	ΔCα − ΔCβ
An	CA	-0.96	52.22	53.18	CB	0.56	19.52	18.96	-1.52
In1	CA	-3.16	58.52	61.68	CB	0.22	38.78	38.56	-3.38
In2	CA	-1.56	60.12	61.68	CB	2.94	41.5	38.56	-4.5
Ln1	CA	-0.23	55.46	55.69	CB	-0.03	42.22	42.25	-0.2
Ln2	CA	-2.86	52.83	55.69	CB	-0.41	41.84	42.25	-2.45
Pn1	CA	-1.60	61.75	63.35	CB	-0.99	30.85	31.84	-0.61
Pn2	CA	-0.33	63.02	63.35	CB	0.19	32.03	31.84	-0.52
Sn	CA	-0.33	58.41	58.74	CB	0.28	64.07	63.79	-0.61
Tn1	CA	0.29	62.55	62.26	CB	0.2	69.9	69.7	0.09
Vn1	CA	-0.67	61.9	62.57	CB	0.8	33.49	32.69	-1.47
Vn2	CA	-3.01	59.56	62.57	CB	0.03	32.72	32.69	-3.04
A1	CA	-0.49	52.69	53.18	CB	0.60	19.56	18.96	-1.09
N1	CA	-0.13	53.42	53.55	CB	0.03	38.99	38.96	-0.16
Y/F	CA	0.05/0.1	58.23	58.18/58.13	CB	-0.38/-1.04	38.88	39.26/39.92	0.43/1.14
G1	CA	0.17	45.54	45.36	CB				0.17
E/Q1	CA	-3.5/-2.76	53.85	57.35/56.61	CB	-0.66/0.14	29.29	29.95/29.15	-2.84/-2.62
E/Q2	CA	-1.62/-0.89	55.73	57.35/56.62	CB	-0.11/0.68	29.84	29.95/29.16	-1.51/-0.21
E/Q3	CA	-1.74/-1.02	55.61	57.35/56.63	CB	2.34/3.12	32.29	29.95/29.17	-4.08/-4.14
H1	CA	-1.14	55.37	56.51	CB	-0.61	29.63	30.24	-0.53
I1	CA	-0.31	61.37	61.68	CB	0.33	38.89	38.56	-0.64
I2	CA	-3.45	58.23	61.68	CB	0.32	38.88	38.56	-3.77
L1	CA	-0.32	55.37	55.69	CB	0.39	42.64	42.25	-0.71
L2	CA	-2.63	53.06	55.69	CB	0.01	42.26	42.25	-2.64
P1	CA	-0.04	63.31	63.35	CB	0.47	32.31	31.84	-0.51
P2	CA	-1.77	61.58	63.35	CB	-0.91	30.93	31.84	-0.86
P3	CA	-0.59	62.76	63.35	CB	2.81	34.65	31.84	-3.40
S1	CA	-0.17	58.57	58.74	CB	0.31	64.10	63.79	-0.48
T1	CA	-0.16	62.10	62.26	CB	0.43	70.13	69.70	-0.59
T2	CA	-2.10	60.16	62.26	CB	0.24	69.94	69.70	-2.34
V1	CA	-0.05	62.53	62.57	CB	0.32	33.01	32.69	-0.36
V2	CA	-2.61	59.96	62.57	CB	0.28	32.97	32.69	-2.89

Figure S1. TEM images of the amelogenin self-assemblies formed at pH 4.5, 5.0 and 5.5 at 37°C. (A) pH 4.5 with 7 days' incubation, (B) pH 5.0 with 32 days' incubation, (C) pH 5.5 with 14 days' incubation.

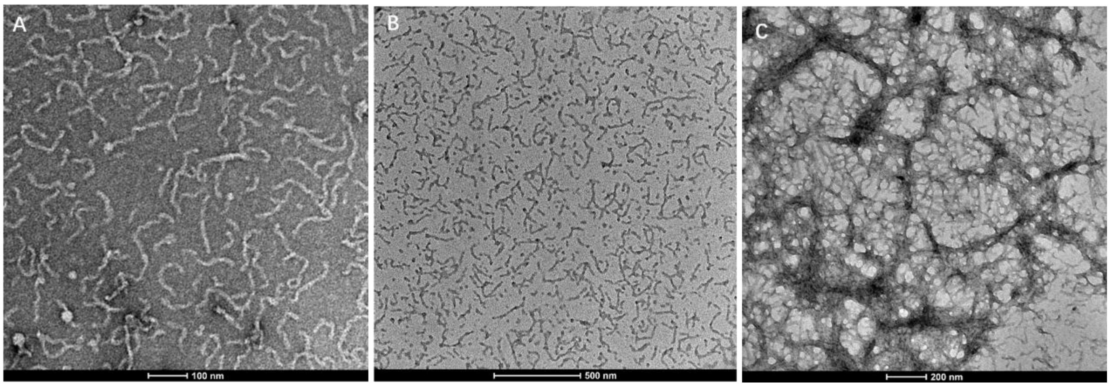

Figure S2. Comparison of the powder X-ray diffractogram of the samples prepared at pH 6.0 with H175 (labeled as pH 6.0) and without H175 (pH 6.0-blank). XRD of octacalcium phosphate (OCP), brushite and hydroxyapatite (HAP) crystals were also shown (data taken from the inorganic crystal structure database OCD-65347-ICSD, brushite-16132-ICSD, HAP-169498-ICSD).

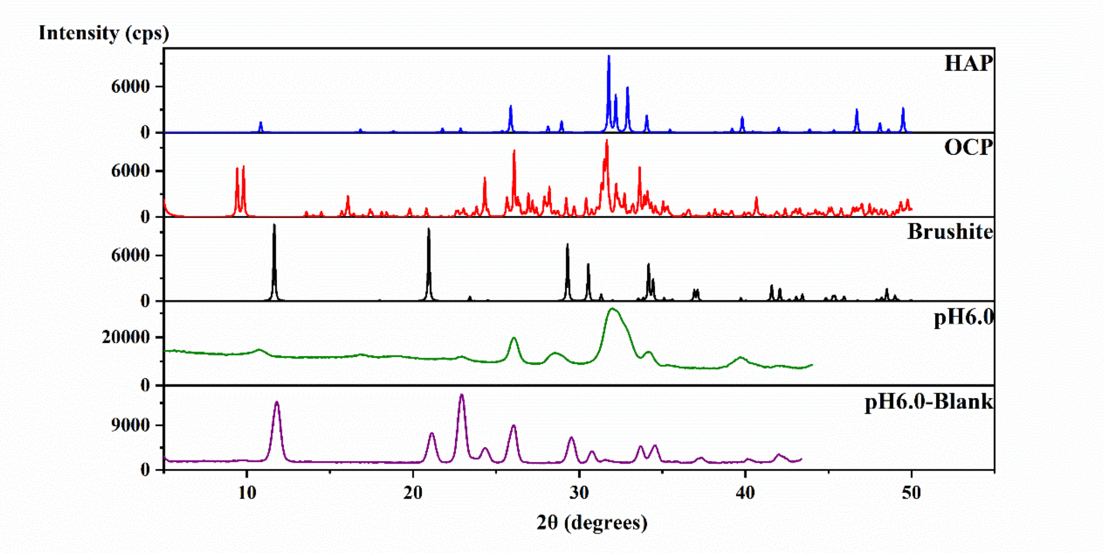

Figure S3. Pulse sequence for ^{1}H-^{31}P HETCOR experiment.

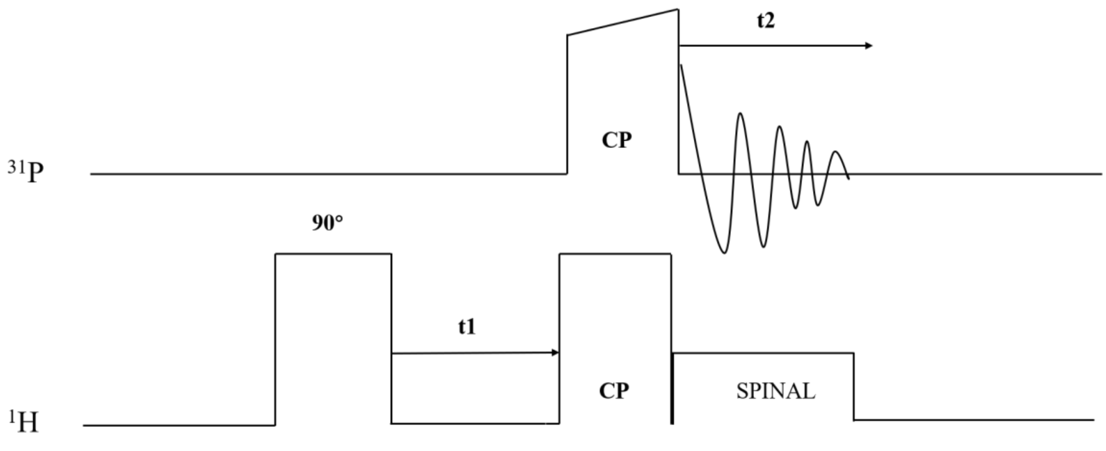

Figure S4. Overlay of 2D ^{13}C-^{13}C correlation spectra for H175 self-assemblies prepared at pH 6.0 (blue) and at pH 4.5 (red).

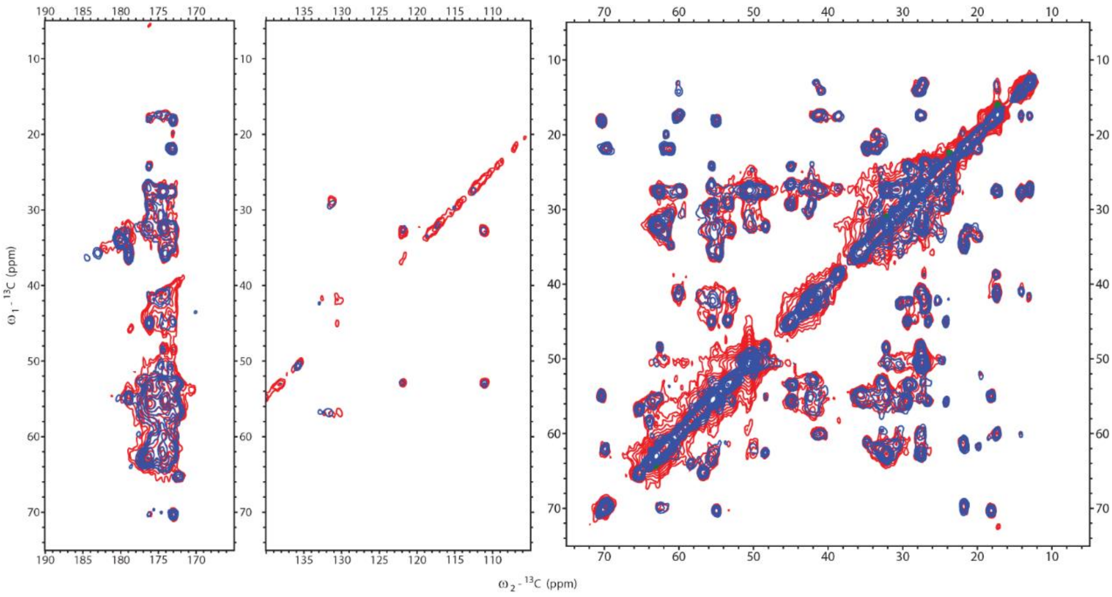

Figure S5. Selective strip plot of 3D NCaCx (blue) and NCoCx (red) spectra to show the sequential assignment. The solid line between (a) and (b) indicates the position of L20Ca and the other line between (c) and (d) represents the position of T21Ca. The ^{15}N chemical shifts labeled corresponds to the residues shown on the top of the strip. L20N at 130.9 ppm, T21N at 114.5 ppm and P22N at 137.6 ppm.

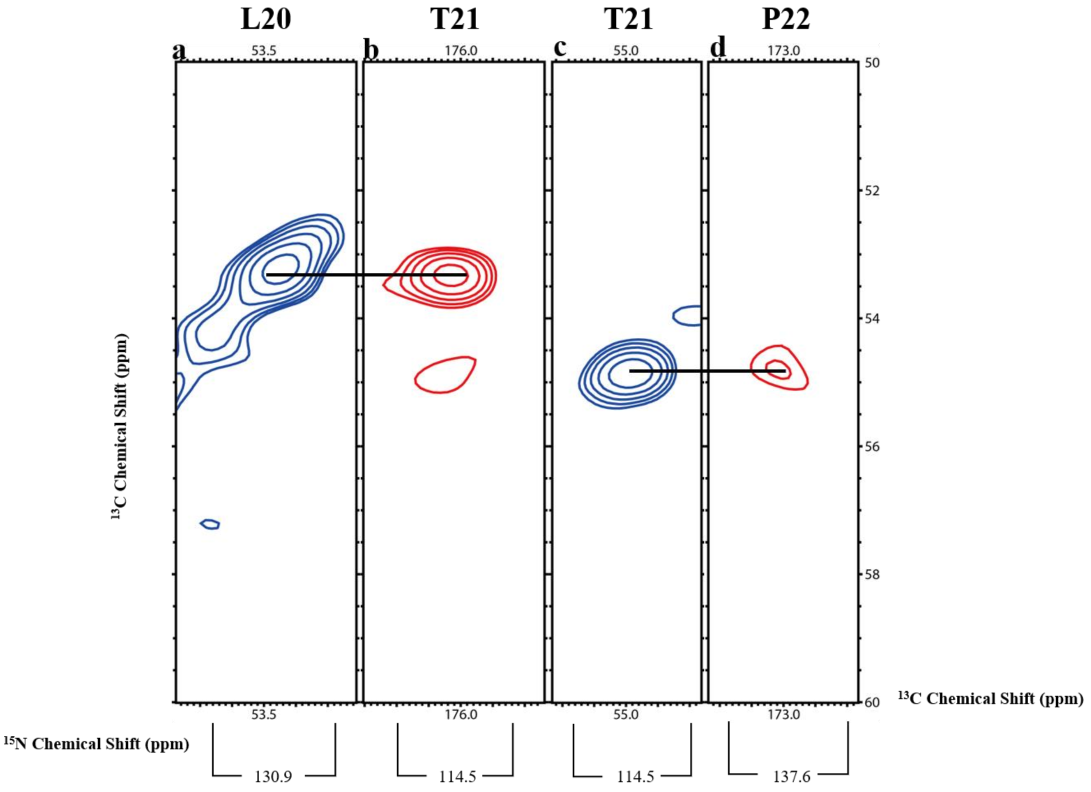

Figure S6. (A) 2D ^{13}C-^{13}C INEPT-TOBSY spectrum of uniformly [^{13}C, ^{15}N]-labeled H175 assemblies prepared at pH 4.5 (B) Overlay of 2D INEPT-TOBSY spectra for the H175 assemblies prepared at pH 6.0 (blue) and pH 4.5 (red). The rectangles highlight some peaks only shown in pH 6.0 spectrum.

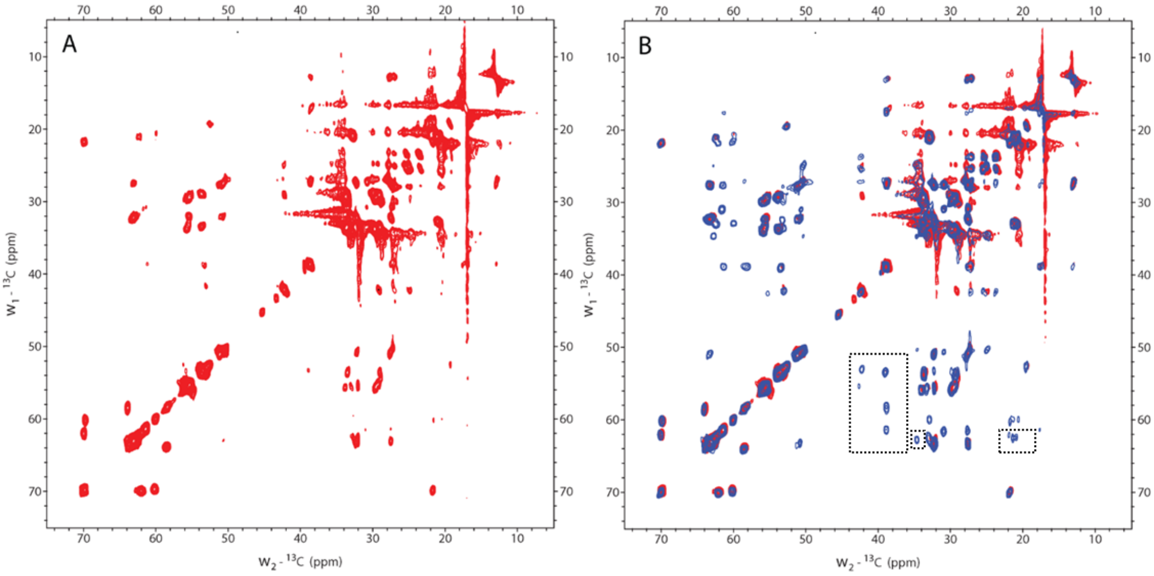

Supplement: Supplementary file 1 [file ijms-21-02946-s001.pdf]
